# Case management vocational rehabilitation for women with breast cancer after surgery: a feasibility study incorporating a pilot randomised controlled trial

**DOI:** 10.1186/1745-6215-14-175

**Published:** 2013-06-14

**Authors:** Gill Hubbard, Nicola M Gray, Dolapo Ayansina, Josie M M Evans, Richard G Kyle

**Affiliations:** 1 Cancer Care Research Centre, School of Nursing, Midwifery and Health, University of Stirling, Highland Campus, Centre for Health Science, Old Perth Road, Inverness IV2 3JH, UK; 2 School of Nursing, Midwifery and Health, University of Stirling, Stirling FK9 4LA, UK; 3 Division of Applied Health Sciences, School of Medicine and Dentistry, University of Aberdeen, Aberdeen, Scotland, UK

**Keywords:** Cancer survivor, Breast cancer, Vocational rehabilitation, Work, Employment, Sickness absence

## Abstract

**Background:**

There is a paucity of methodologically robust vocational rehabilitation (VR) intervention trials. This study assessed the feasibility and acceptability of a VR trial of women with breast cancer to inform the development of a larger interventional study.

**Methods:**

Women were recruited in Scotland and randomised to either a case management VR service or to usual care. Data were collected on eligibility, recruitment and attrition rates to assess trial feasibility, and interviews conducted to determine trial acceptability. Sick leave days (primary outcome) were self-reported via postal questionnaire every 4 weeks during the first 6 months post-surgery and at 12 months. Secondary outcome measures were change in employment pattern, quality of life and fatigue.

**Results:**

Of the 1,114 women assessed for eligibility, 163 (15%) were eligible. The main reason for ineligibility was age (>65 years, *n* = 637, 67%). Of those eligible, 111 (68%) received study information, of which 23 (21%) consented to participate in the study. Data for 18 (78%) women were analysed (intervention: *n* = 7; control: *n* = 11). Participants in the intervention group reported, on average, 53 fewer days of sick leave over the first 6 months post-surgery than those in the control group; however, this difference was not statistically significant (*p* = 0.122; 95% confidence interval −15.8, 122.0). No statistically significant differences were found for secondary outcomes. Interviews with trial participants indicated that trial procedures, including recruitment, randomisation and research instruments, were acceptable.

**Conclusions:**

Conducting a pragmatic trial of effectiveness of a VR intervention among cancer survivors is both feasible and acceptable, but more research about the exact components of a VR intervention and choice of outcomes to measure effectiveness is required. VR to assist breast cancer patients in the return to work process is an important component of cancer survivorship plans.

**Trial registration:**

ISRCTN29666484

## Background

Work is beneficial for physical and mental health [1]. Returning to work following diagnosis and treatment for cancer is a public health concern because being in paid employment is associated with improved quality of life [2-5] and perceived by cancer survivors as a means of re-gaining a sense of normality, self-concept and identity [6-11]. In the UK around 90,000 people of working age are diagnosed with cancer annually [12]. Breast cancer is the most common female cancer worldwide [13] and many women diagnosed with the disease will be in paid employment at the time of their diagnosis.

Studies about breast cancer and employment suggest that the overwhelming majority of women with breast cancer return to work (rates above 80% reported) [14-16]. Yet, a review of 26 articles about cancer survivors, which found that return to work varies according to the type of cancer, reports that there is an increased risk of unemployment for breast cancer survivors versus control participants (35.6% vs. 31.7%; pooled RR, 1.28; 95% CI, 1.11-1.49) [17]. Moreover, a recent study conducted in Germany of 227 breast cancer patients versus 647 comparison participants reported that 6 years after surgery, the probability of returning to work was still only half as high among breast cancer survivors than among controls [18].

The duration of work absence varies, suggesting that some women find it harder returning to work than others [14-16]. A review found the average length of sick leave for a person treated for cancer to be 151 missed days from work [19]. A study conducted in North America found that women treated for breast cancer missed an average of 44.5 days from work within the first 6 months following diagnosis; the median days missed was 22 [20]. Length of work absence is important because sickness absence is a risk factor for permanently leaving the workforce [21]. A survey of 267 cancer survivors (48% were breast cancer survivors) in England found that the length of sick leave was significantly related to return to work within 18 months of cancer diagnosis [odds ratio 1.68, 95% confidence interval (CI) 1.23-2.28] [22].

Treatment modality may be one contributing factor to work absence duration. A recent study found that women who had undergone mastectomy had longer sickness absence compared to women who had undergone breast-conserving surgery [22]. Other studies report a negative impact of chemotherapy on duration of work absence in breast cancer survivors [20,23,24]. A systematic review of 28 cohort studies about predictors of return to work and employment in cancer survivors with different diagnoses found that heavy work, chemotherapy, older age, low education and low income were negatively associated with return to work [25].

Studies show that women with breast cancer compared to cancer-free controls will, over time, experience permanently reduced work ability [26,27]. A cross-sectional study found that women with breast cancer had significantly lower work productivity than their peers who had no personal experience of cancer and that fatigue was associated with lower work productivity [28]. Similarly, a recent study of breast cancer survivors who were diagnosed with stage 0 ductal carcinoma in situ or stage I, II or III breast cancer found a mean reduction in productivity of 3.1% below the healthy worker norm, even at 3 years post treatment; after controlling for stage, fatigue and hot flashes were each associated with work performance losses of 1.6% (*p* = 0.05) and 2.2% (*p* < 0.001), respectively [29].

Whether returning to work is a positive experience for cancer survivors depends on a range of factors including good organisational support, such as an employer’s willingness to make adjustments to the workplace and job role, and informal personal and emotional support from colleagues [11]. A focus group study of women with breast cancer found that disclosure of their cancer in the workplace in particular could be distressing [30]. Other research suggests that a minority of cancer survivors experience job discrimination [30,31] and become involved in disputes about terms of employment [32]. Other reasons why work experience may prove difficult include lack of understanding of the role of occupational health by treating doctors [33].

A consequence of over a decade of cancer and employment research is growing interest in vocational rehabilitation (VR) services for cancer survivors [34]. However, the availability of rehabilitation services varies considerably [35]. A recent review found only 19 work-related interventions for cancer survivors and only 3 of these interventions focussed primarily on improving work outcomes [36]. Another review noted the lack of services in the UK designed to help people with cancer remain in or return to work [37]. Systematic reviews of VR interventions for cancer survivors have noted a paucity of methodologically robust interventions [36,38-40]. A recent controlled trial involving 72 cancer patients suggests that high-intensity physical training is useful for working cancer patients; patients in the intervention group showed significantly less reduction in working hours per week [−5.0 h/week vs. -10.8 h/week (*P* = 0.03)], and on long-term follow-up, 78% of the participants from the intervention group versus 66% from the control group had returned to work on the pre-diagnosis level of working hours (*P* = 0.18) [41]. A nurse-led intervention whereby patients with breast cancer were encouraged to return to work and become socially active, and were counselled on feelings, found that 12 to 18 months after surgery, those who were helped by the nurse had greater social recovery, return to work and adaptation to breast loss than those without the nurse’s support [42]. In a randomised trial, patients with breast cancer receiving information and performing physical training supplemented by coping skills training provided by an oncology nurse who specialised in psychosocial matters had improved return-to-work outcomes, but no statistically significant differences were observed when those patients were compared with controls who received either a single information session or no intervention [43].

Thus, there is only limited evidence of the effectiveness of rehabilitation programmes on return to work for cancer survivors [44,45]. To our knowledge, this is the first article reporting the use of a general as opposed to cancer-specific VR service for cancer patients.

### AIM

The aim of this study was to assess the feasibility and acceptability of an existing case management VR service for women with breast cancer to inform the development of a larger randomised controlled trial (RCT). Hence, this feasibility study incorporated a pilot RCT with women with breast cancer following surgery. Although hypothesis testing must proceed cautiously in a feasibility study, it was anticipated that participants referred to the VR service would experience fewer days off work due to sickness in the first 6 months post-surgery (primary outcome), lower levels of fatigue and increased quality of life (secondary outcomes). This article reports the feasibility and acceptability of this trial and VR services among women with breast cancer, as well as trial outcomes at 6- and 12-month follow-up.

## Methods

### Study design

The design of this feasibility study incorporating an interventional two-arm pilot randomised controlled trial has been previously described [46]. Trial procedures are therefore only described briefly below, with particular attention paid to deviations from protocol. Three changes were made to the protocol during the pilot RCT in response to lower than expected recruitment rates in order to prevent the RCT failing to recruit. These changes were:

1. Inclusion of women with ductal carcinoma in situ (DCIS) (from 1 June 2011);

2. Extension of the recruitment period by 6 months (from 1 June 2011);

3. Inclusion of women employed in companies with more than 250 employees (from 1 August 2011).

Changes to eligibility criteria resulted in the recruitment of an additional four RCT participants who would previously have been excluded. These included two women diagnosed with DCIS and two individuals employed in companies with more than 250 employees. The study was closed to recruitment on 29 December 2011 and concluded, as planned, on June 12 2012.

### Participants

Eligible patients were women: (1) aged between 18 and 65 years; (2) in paid employment or self-employed; (3) living or working in Lothian or Tayside, Scotland, UK; (4) diagnosed with an invasive breast cancer tumour or ductal carcinoma in situ (DCIS); (5) treated first with surgery. Breast care nurses (BCN) assessed eligibility of patients attending hospital in the preoperative phase.

### Recruitment

Recruitment was conducted over a 14-month period between September 11 2010 and 29 December 2011.

### Settings

Participants were recruited from three hospitals in two NHS Boards in Scotland [Perth Royal Infirmary (PRI), and Ninewells Hospital, Dundee (NHS Tayside); Western General Hospital (WGH), Edinburgh (NHS Lothian)]. Due to variation in local clinical processes, service size and workforce capacity, the recruitment process and timeframe differed in each of the three hospitals.

### Recruitment Procedure

Trial eligibility was assessed through a two-stage process. First, clinical eligibility criteria were assessed by either clinical teams from surgical lists (NHS Tayside) or at a weekly multi-disciplinary team (MDT) meeting (NHS Lothian). Second, employment eligibility criteria were assessed by BCNs. Eligible women were given a study information pack at a routine appointment with their BCN and returned written informed consent to either their BCN or the study researcher (RGK) by post.

### Intervention

The Scottish Centre for Healthy Working Lives established ‘Working Health Services’ (WHS), a pilot VR service, in NHS Tayside and NHS Lothian in 2008 and 2009, respectively. WHS provides fast-track support to people to remain in or return to work following a period of injury or illness who are employed in companies with less than 250 employees where occupational health services are not routinely available. WHS adopts a biopsychosocial model and a multi-disciplinary approach whereby case management is used to assess individuals’ needs to enable work retention or return through signposting or direct referral to a range of supportive services according to need, such as physiotherapy, occupational therapy, occupational health nurse, occupational health doctor, counsellor/psychological therapy and complementary therapy. However, WHS is not cancer-specific and prior to the commencement of this study only a few individuals with cancer had been referred to WHS in both Tayside and Lothian. The purpose of this study was therefore to assess the feasibility of referral of cancer patients to an existing case management VR service and, specifically, the acceptability of WHS for women with breast cancer.

Thus, the trial intervention was referral to WHS in either Tayside or Lothian. Patients recruited from PRI and Ninewells, Dundee, were randomised to receive referral to WHS Tayside; individuals enrolled from WGH were referred to WHS Lothian. All participants allocated to the intervention arm of the trial were contacted by WHS within 10 days following return of the baseline questionnaire to the study researcher (RGK). As per usual WHS practice, participants were allocated a ‘case manager’ who conducted a telephone assessment of supportive care needs to facilitate work retention or return. Based on this assessment (where appropriate) individuals were signposted or referred to relevant services that could support patients with cancer-related and treatment side effects (e.g., fatigue, mood changes) as well as job-related issues (e.g., liaison with employers to enable work adjustments such as changes to hours worked or job role) in order to decrease duration of sickness absence or increase overall quality of (work) life. Hence, reflecting the personalised nature of the case management VR approach, each individual could receive a different (combination of) intervention(s), which precluded exogenous standardisation.

### Usual care

Usual care following surgery involved no formal employment support. However, participants in both arms of the trial received a copy of the booklet *Work and Cancer* published by Macmillan Cancer Support [47].

### Measures

The primary objective of the study was to assess the feasibility and acceptability of trial processes and the intervention to inform a larger RCT of the effectiveness of a case management VR service for patients with cancer. Data were therefore collected on eligibility, recruitment and attrition rates to assess trial feasibility. Moreover, a qualitative evaluation was conducted to determine trial acceptability. This evaluation involved 11 semi-structured interviews with trial participants (*n* = 6), breast cancer clinical nurse specialists (*n* = 3) and VR service case managers (*n* = 2). Interviews with trial participants were conducted, as planned, at 6-month follow-up. However, due to the 6-month extension of the recruitment period, only 12 women were entered into the trial at this time, of whom half were interviewed. Thus, interviewees were representative of trial participants prior to the change in eligibility criteria. Interviews were audio recorded and transcribed verbatim. Transcripts were analysed by identifying similarities/differences in responses between respondents to structured questions about trial acceptability and feasibility. Frequency of particular responses was noted and quotations selected (where appropriate) to illustrate specific points. A structured questionnaire was used to collect baseline data on date of birth, postcode, total household income, clinical diagnosis and co-morbidities.

In addition, the following primary and secondary trial outcomes were assessed. There were no changes to these outcomes after commencement of the trial.

#### Primary outcome (self-reported sickness absence)

The primary outcome was number of days off work due to ill health within the first 6 months after surgery. Sick leave days were self-reported via postal questionnaire every 4 weeks during the first 6 months post-surgery, which is known to be a robust approach where recall is limited to 2 to 4 weeks [48,49]. In addition, the duration of sick leave in the 4 weeks before the date of 12-month follow-up was also measured by self-report postal questionnaire. Because it is likely to be difficult to directly attribute a day of sickness absence to breast cancer and its treatments (particularly through a self-report measure), all sickness absence, irrespective of its perceived or actual relationship with cancer, was measured.

#### Secondary outcomes

Secondary outcome measures were change in employment pattern, health-related quality of life (HRQoL) and fatigue between enrolment and 6- and 12-month follow-up. Secondary outcomes were assessed by self-report postal questionnaire at trial enrolment, 6- and 12-month follow-up (between 17 May 2011 and 19 May 2012, and 17 November 2011 and 19 November 2012, respectively).

#### Employment pattern

A non-validated questionnaire measured change in employment pattern including the following indicators: left or remained in paid employment, job role and hours worked.

#### Quality of life

The Functional Assessment of Cancer Therapy-Breast Cancer (FACT-B) Version 4 was used to assess breast cancer-related quality of life (QoL) [50]. The FACT-B is a 37-item self-report questionnaire that evaluates several QoL domains: physical, social/family, emotional and functional well-being. In addition, a ten-item breast cancer subscale (BCS) is specific to the experiences of women living with breast cancer and the symptoms and side effects of treatment and includes items on anxiety, pain and body image. Participants complete the questionnaire in terms of the past 7 days and each item is scored on a 5-point Likert scale that varies from 0 (not at all) to 4 (very much). Negatively phrased questions are reversed prior to analysis and scores are summed for each domain; a higher score indicates higher well-being [50]. Domain scores vary from 0 to 28 for the physical, social/family and functional well-being domains; 0 to 24 for the emotional well-being domain and 0 to 40 for the BCS. The scores of the four well-being domains are summed to calculate the Functional Assessment of Cancer Therapy-General (FACT-G) score and total scores of the FACT-G and BCS are summed to calculate a FACT-B score.

#### Fatigue

The Functional Assessment of Chronic Illness Therapy-Fatigue Scale (FACIT-Fatigue) was used to assess specific functional and physical aspects of fatigue associated with breast cancer diagnosis and treatment. This 13-item subscale has been determined to be a reliable and valid stand-alone measure of fatigue [51]. In common with the FACT-B, items are scored on a 5-point Likert scale and relate to the past 7 days. FACIT-Fatigue scores vary from 0 to 52 and negatively worded items are reversed before analysis so that higher scores represent better self-reported health [51].

### Sample size

Based on local Tayside and Lothian diagnosis and surgical data obtained from 2008, estimated employment rates and a conservative recruitment rate of between 50% and 60%, it was initially estimated that between 66 and 79 patients would be recruited to the study over a 6-month period [46]. Sample size calculations based on the primary outcome measure reported in the protocol [46] indicated that, assuming a mean of 180 days absent from work in the control group and an estimated standard deviation of 100 days, a sample size of 70 (35 in each group) would be able to detect a reduction in number of days absent from work from 180 to 110 days with a 5% level of significance and 80% power. However, this recruitment target was not met and consequently the study was underpowered to identify statistically significant differences in the primary and secondary outcome measures between the intervention and control groups.

### Randomisation

The allocation sequence was generated from a Bernoulli probability distribution with a specified probability of 0.5, which ensured participants had an equal chance of being in either group. Participants were randomly assigned to the intervention and usual care arm with a 1:1 allocation ratio. A separate sequence was used for each NHS Board (analogous to the VR service to which participants in the intervention arm are referred) to ensure that there was an even distribution of participants to the intervention and usual care groups in each NHS Board. The allocation sequences were concealed from the researcher (RGK) responsible for recruiting and collecting data from participants. The trial statistician (DA) provided the allocation sequences to a research administrator in the Cancer Care Research Centre at the University of Stirling who was not involved in the process of random number generation, data collection or analysis. This administrator assigned participants to the intervention and usual care groups and referred participants in the intervention arm to the VR service.

### Blinding

Participants became aware of their group allocation at the point at which they did or did not receive a referral to the VR service. Thus, it was not possible to blind patients to their group allocation. However, the randomisation procedure ensured that researchers involved in data collection or outcome assessment did not know participants’ group allocation.

### Statistical methods

Data analysis was conducted using SPSS (version 19) [52]. Baseline characteristics are reported as mean and standard deviation for continuous data and *n* (%) for categorical data. Differences between the intervention and control groups for the primary outcome measure were tested using an independent samples *t*-test as per protocol [46]. Due to the small number of participants recruited to the trial, per protocol analysis of secondary outcomes using analysis of covariance (ANCOVA), adjusting for baseline values was not conducted following advice from the trial statistician (DA). Secondary outcomes were therefore also assessed using independent samples t-tests. The significance level was set at 0.05 as per protocol [46].

### Ethical approval

The study protocol was approved by NHS Tayside Committee on Medical Research Ethics A (ref.: 10/S1401/15) and the research ethics committee of the University of Stirling. Research Governance approval was also obtained from NHS Lothian and NHS Tayside. Informed written consent was obtained from participants.

## Results

### Participant flow (trial feasibility)

**Figure 1:**
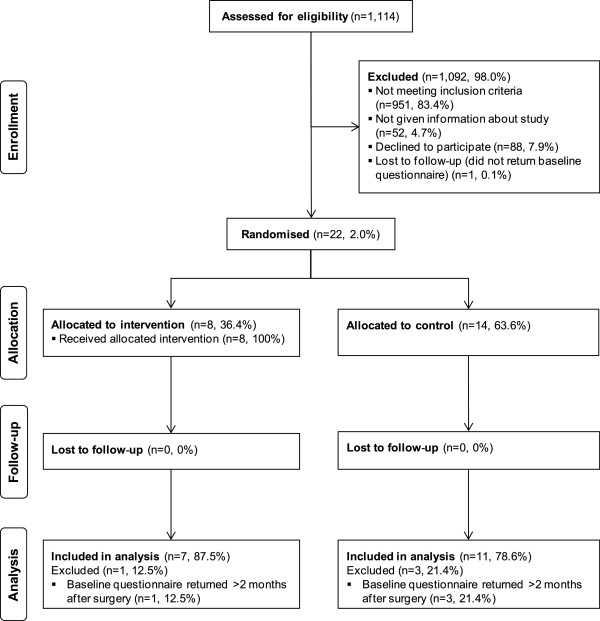
CONSORT pilot RCT participant flow diagram.

Participant flow through the pilot RCT is illustrated in Figure 1. Of the 1,114 women assessed for eligibility 951 (85.4%) were ineligible because of age > 65 (*n* = 637, 67%); being unemployed (*n* = 115, 12.1%); being employed in companies with >250 employees [assessed in WGH only to 31 July 2011 because of the recruitment process (see above), *n* = 83, 8.7%]; surgery not being the first treatment (WGH only, *n* = 64, 6.7%); non-invasive disease (WGH only to 31 May 2011, *n* = 31, 3.3%); resident/work outside the VR service area (*n* = 21, 2.2%). Of the 163 (14.6%) women eligible for inclusion, 52 (31.9%) were not given information about the study for the following reasons: failure in the distribution process (e.g., nurse forgetfulness; *n* = 27, 51.9%); information declined by prospective participant because of perceived relevance (*n* = 15, 28.8%); BCN considered study inappropriate because of psychological/emotional distress (*n* = 5, 9.6%) or personal/social circumstances (*n* = 2, 3.8%); communication difficulties (*n* = 3, 5.8%). Of the 111 (68.1%) women who did receive study information, 23 (20.7%) consented to participate in the study. One woman (4.3%) did not return a baseline questionnaire despite repeat reminders and data for four women (17.4%) could not be analysed because more than 2 months had elapsed between the date of recruitment and receipt of the baseline questionnaire. Data for 18 (78.3%) women were analysed (intervention: *n* = 7, control: *n* = 11). Data for two women, both randomly allocated to the control group, could not be included in analysis of the secondary quality of life and fatigue outcomes as the number of missing items on returned questionnaires was greater than that permissible by FACT analysis procedures [50].

### Sample characteristics

Sociodemographic and clinical characteristics of participants included in the analysis are shown in Tables 1 and 2. The mean age of participants was 50.5 (SD = 5.5) and most women were diagnosed with stage II breast cancer (44.4%), followed by stage III (27.8%). Two-thirds (66.7%) stated that they had a co-morbidity. Most women (61.1%) worked full time and reported an annual household income of between £20,000 and £39,999. The mean number of hours worked each week was 32.5 (SD = 10.2).

**Table 1:** Sample characteristics at baseline assessment

	**All (*n *= 18)**		**Intervention (*n *= 7)**		**Control (*n *= 11)**	
	***n***	**%**	***n***	**%**	***n***	**%**
**Treatment hospital**						
Perth Royal Infirmary	7	38.9	3	42.9	4	36.4
Ninewells Hospital	7	38.9	3	42.9	4	36.4
Western General Hospital	4	22.2	1	14.3	3	27.3
**Vocational rehabilitation service area**						
Lothian	4	22.2	1	14.3	3	27.3
Tayside	14	77.8	6	85.7	8	72.7
**Age (at surgery)**						
30-39	1	5.6	1	14.3	0	0
40-49	6	33.3	2	28.6	4	36.4
50-59	10	55.6	3	42.9	7	63.6
60-65	1	5.6	1	14.3	0	0
Mean [SD]	50.5	[5.5]	49.7	[7.6]	51.0	[3.9]
Median	50		50		51	
Min	37		37		45	
Max	63		63		59	
**Breast cancer diagnosis**						
Stage 0 (DCIS)	2	11.1	1	14.3	1	9.1
Stage I	3	16.7	2	28.6	1	9.1
Stage II	8	44.4	3	42.9	5	45.5
Stage III	5	27.8	1	14.3	4	36.4
**Co-morbidities**						
Yes	6	33.3	2	28.6	4	36.4
No	12	66.7	5	71.4	7	63.9
**Employment status**						
Full time	11	61.1	6	85.7	5	45.5
Part time	7	38.9	1	14.3	6	54.5
**Hours worked per week**						
Less than 20	2	11.1	1	14.3	1	9.1
20-39	5	27.8	0	0	5	45.5
30-39	9	50	4	57.1	5	45.5
40-49	0	0	0	0	0	0
50 or more	2	11.1	2	28.6	0	0
Mean [SD]	32.5	[10.0]	37.1	[11.7]	29.6	[8.0]
Median	36.3		36.5		28	
Min	15		15		18	
Max	50		50		38	
**Job role**						
Manager	7	38.9	3	42.9	4	36.4
Foreman or supervisor	2	11.1	2	28.6	0	0
Other employee	9	50.0	2	28.6	7	63.6
**Employment sector**						
Healthcare	3	16.7	0	0	3	27.3
Education	4	22.2	2	28.6	2	18.2
Service	3	16.7	1	14.3	2	18.2
Social care	2	11.1	1	14.3	1	9.1
Retail	2	11.1	2	28.6	0	0
Entertainment	1	5.6	1	14.3	0	0
Civil service	2	11.1	0	0	2	18.2
Media	1	5.6	0	0	1	9.1
**Household income (£)**						
Less than 20,000	2	11.1	0	0	2	18.2
20,000 - 29,999	5	27.8	4	57.1	1	9.1
30,000 - 39,999	6	33.3	1	14.3	5	45.5
40,000 - 49,999	1	5.6	1	14.3	0	0
50,000 and above	3	16.7	1	14.3	2	18.2
Not reported	1	5.6	0	0	1	9.1

**Table 2:** Participants’ treatment modalities during trial

	**All (*n *= 18)**		**Intervention (*n *= 7)**		**Control (*n *= 11)**	
**Treatment modality**	***n***	**%**	***n***	**%**	***n***	**%**
Surgery only	3	16.7	2	28.6	1	9.1
Surgery + RTx^1^	7	38.9	2	28.6	5	45.5
Surgery + CTx^2^ + RTx	8	44.4	3	42.9	5	45.5

Notes: ^*1*^*RTx* radiotherapy, ^*2*^*CTx* chemotherapy.

### Intervention

Case management interventions received by participants in the intervention group are shown in Table 3. Due to the recruitment process all women received at least one telephone call from a case manager. Four women (57.1%) received only telephone support. Participants had 27 telephone contacts with a case manager (mean = 3.9). Three women (42.9%) also had a face-to-face meeting with a case manager, which for one individual also involved their Human Resources (HR) advisor at work. These three participants had a total of seven face-to-face meetings with a case manager (mean = 2.3). Two individuals (28.6%) were referred to another service via the VR service. One woman was referred to Maggie’s Cancer Caring Centre for welfare and benefits advice and for assistance with an application for financial aid from Macmillan Cancer Support. Another participant was referred to an employment rights service for advice around phased return to work.

**Table 3:** VR service case management interventions

	**Contacts**		
**Participant**	**Telephone**	**Face-to-face**	**Referrals**
1	1	0	0
2	1	0	0
3	2	0	0
4	2	1	0
5	10	3	0
6	2	0	1
7	9	3	1
Total	**27**	**7**	**2**

### Outcome measures

Trial outcome data are shown in Tables 4 and 5.

**Table 4:** Pilot RCT outcome measures (6-month follow-up)

	**6-month follow-up**									
	**All**		**Intervention**		**Control**			**95% CI**		
**Outcome**	**Mean**	**[SD]**	**Mean**	**[SD]**	**Mean**	**[SD]**	**Mean diff.**	**Lower**	**Upper**	***p* value**
*Primary*										
Sick leave (days)	87.8	[70.4]	55.4	[52.4]	108.5	[74.7]	53.1	−15.8	122.0	0.122
*Secondary*										
FACT-B [0–144]	103.4	[20.0]	109.0	[17.9]	98.9	[21.4]	10.1	−31.7	11.5	0.333
Physical (PWB) [0–28]	22.4	[5.4]	23.1	[3.9]	21.9	[6.5]	1.2	−7.2	4.8	0.680
Social/family (SWB) [0–28]	20.1	[7.5]	19.7	[7.5]	20.5	[8.0]	−0.8	−7.6	9.2	0.841
Emotional (EWB) [0–24]	18.9	[3.9]	19.7	[2.4]	18.3	[4.9]	1.4	−5.4	2.7	0.471
Functional (FWB) [0–28]	18.9	[6.0]	20.1	[7.9]	18.0	[4.2]	2.1	−8.7	4.4	0.494
Breast cancer sub-scale (BCS) [0–36]	22.9	[5.5]	26.4	[4.7]	20.2	[4.7]	6.2	−11.2	−1.1	**0.020**
FACT-B Trial Outcome Index (TOI) [0–92]	64.3	[13.1]	69.6	[11.7]	60.1	[13.1]	9.5	−23.1	4.1	0.155
FACIT-Fatigue [0–52]	35.5	[13.8]	35.7	[13.5]	35.4	[14.9]	0.3	−15.7	15.2	0.971

**Table 5:** Pilot RCT outcome measures (12-month follow-up)

	**All**		**Intervention**		**Control**			**95% CI**		
**Outcome**	**Mean**	**[SD]**	**Mean**	**[SD]**	**Mean**	**[SD]**	**Mean Diff.**	**Lower**	**Upper**	**p value**
*Primary*										
Sick Leave (Days)	2.9	[4.4]	1.6	[3.6]	3.6	[4.8]	2.0	−3.4	7.3	0.441
*Secondary*										
FACT-B [0–144]	110.0	[18.9]	113.7	[18.5]	107.1	[19.8]	6.6	−27.3	14.2	0.510
Physical (PWB) [0–28]	24.3	[4.0]	25.0	[1.4]	23.8	[5.2]	1.2	−5.6	3.2	0.560
Social/family (SWB) [0–28]	20.0	[6.1]	21.3	[5.4]	18.9	[6.7]	2.4	−9.1	4.2	0.450
Emotional (EWB) [0–24]	19.1	[3.9]	19.7	[4.6]	18.7	[3.3]	1.0	−5.3	3.2	0.606
Functional (FWB) [0–28]	21.3	[5.5]	20.7	[6.9]	21.8	[4.4]	−1.1	−5.0	7.1	0.713
Breast cancer sub-scale (BCS) [0–36]	25.3	[4.3]	26.9	[4.5]	24.0	[3.9]	2.9	−7.4	1.6	0.184
FACT-B Trial Outcome Index (TOI) [0–92]	70.9	[10.9]	72.7	[10.0]	69.6	[11.9]	3.1	−15.1	8.9	0.590
FACIT-Fatigue [0–52]	41.2	[9.4]	41.0	[9.5]	41.3	[9.9]	−0.3	−10.2	10.8	0.947

#### Primary outcome (self-reported sickness absence)

Participants in the intervention group reported 53.1 fewer days of sick leave over the first 6 months post-surgery than those in the control group, equivalent to 1.7 months. Although substantial, this difference was not statistically significant (*p* = 0.122; 95% CI −15.8, 122.0) (Table 4).

At 12-month follow-up the intervention group reported 2 fewer days of sick leave during the preceding 4 weeks than those in the control group, although, again, this difference was not statistically significant (Table 5).

#### Secondary outcomes (employment patterns, quality of life and fatigue)

##### Employment patterns

No participants retired, left paid employment or changed jobs during the trial. All participants had the same job role at 12 months as they had reported before their cancer diagnosis. Four participants (57.1%) in the intervention group and three participants (27.3%) in the control group reported a change in hours worked each week during the trial. However, no discernible difference in the pattern of changes to working hours was found between the intervention and control groups.

##### Quality of life

Participants in the intervention group reported higher overall quality of life at 6-month follow-up (FACT-B: 109.0, SD = 17.90) than those in the control group (98.9, SD = 21.39). However, this difference was not statistically significant (*p* = 0.333). Measures of physical, emotional and functional well-being were all higher in the intervention group than in the control group, although differences were not statistically significant. Social/family well-being was lower in the intervention group at 6-month follow-up, but not statistically significantly. Participants in the intervention group reported a statistically significantly higher score on the breast cancer subscale (BCS) than those in the control group (intervention: 26.4 [SD = 4.65], control: 20.2 [SD = 4.68], *p* = 0.020; Table 4).

A similar pattern was found at 12-month follow-up with participants in the intervention group reporting higher levels of overall quality of life (FACT-B), physical and emotional well-being, and a higher BCS score. Social/family well-being was, however, higher in the intervention group at 12-month follow-up. None of the differences at 12-month follow-up were statistically significant (Table 5).

##### Fatigue

There were only very small differences in self-reported levels of fatigue (FACIT-Fatigue) between the intervention and control groups at both 6- and 12-month follow-up; these were not statistically significant (Tables 4 and 5).

### Trial acceptability

#### Trial participants

Interviews with trial participants (*n* = 6) indicated that trial procedures, including recruitment, randomisation and research instruments, were acceptable. It was clear to participants why and how they had been recruited to the trial and the initial approach about study involvement through a BCN was considered appropriate. Research instruments were not deemed to be overly burdensome in terms of either completion time or content (e.g., “I didn’t find it a long questionnaire, the questions were brief too”). Typically, baseline and 6- and 12-month follow-up questionnaires took 15–20 min to complete and each sick leave questionnaire was completed in 5 min. Completion of the sick leave questionnaire every 4 weeks was considered appropriate as days absent from work could be more easily recalled and checked against calendar or diary entries over this period. Individual questions were considered to be easily understandable and not excessively intrusive (e.g., “I don’t remember thinking: ‘How dare they ask me that’”). However, some individuals (*n* = 2) did indicate that completing the QoL (FACT) questionnaire in terms of the last 7 days, as instructed, was “tricky” because of daily fluctuation in mood and symptoms (e.g., “Some days you feel fantastic and the next day you feel rubbish so it’s always quite difficult to fill those in because you’re like: ‘Well, today I feel great, but yesterday I felt horrible and who knows what tomorrow holds?’”). Another individual noted that she felt that the QoL questionnaire “had been written by someone who hasn’t been through cancer and if they had been through cancer they would have worded some of the questions slightly differently”. Thus, additional questions on symptoms and timing/frequency of symptom exacerbation were requested. Receipt and return of research instruments by post was deemed appropriate, although all participants (*n* = 6) indicated that email or online completion would also have been appropriate. Participants were content with their group allocation and this contentment was expressed in relation to general appeals to altruistic motivations that underpinned trial participation (e.g., “If it means taking a few minutes every now and again to fill in a form that can help somebody else further down the line then everybody should be doing it”).

#### Breast Care Nurses (BCN)

Breast care nurses (*n* = 3) indicated that the main reasons for not providing information to women were: heightened anxiety among prospective participants; information overload and clinical prioritisation at the point on the patient pathway designated for recruitment; limited time for conversations with women at the time of recruitment; nurse forgetfulness; nurses’ perceptions about trial relevance for prospective participants; and competition between trials and perceived importance of a VR intervention study in relation to other open trials among clinicians. Timing of recruitment was noted by nurses as a particular challenge as it was a point in the patient pathway of heightened cancer worry and information burden. However, nurses also suggested that an alternative recruitment point later in the patient pathway had potential to exclude individuals for whom employment difficulties were experienced earlier in the pathway, such as self-employed people or individuals on short-term contracts. An alternative recruitment procedure was therefore suggested that relied on administrative rather than clinical staff to retrospectively identify patients and send study information by post. However, nurses noted that a challenge of this approach was that patients’ current employment status was not currently routinely recorded and that this would have to be addressed to facilitate this alternative recruitment procedure.

#### VR service staff

VR service staff (*n* = 2) suggested that although the initial contact between trial participants and the service differed from usual practice (i.e., participants were approached by the VR service rather than self-referral), the process from that point did not differ between trial participants and other clients. However, case managers did note that for those individuals referred to the service via the trial, they did not perceive their work to be case management because of its light-touch nature. Additionally, VR staff expressed disappointment that nurses’ perceptions of the level of anxiety and distress among prospective participants had prevented inclusion of some women in the trial and suggested that their role could have contributed to the alleviation of anxiety. Thus, VR staff indicated that further education was required for clinical staff about the role of the VR service and, conversely, also recognised that VR staff needed additional training from clinical staff about the specific needs of cancer patients to better support these individuals.

## Discussion

The importance of feasibility and pilot trials of pragmatic complex interventions [53] prior to the conduct of effectiveness trials is recognised [54-56] although these studies are under-reported, which means that few opportunities exist to share learning from important preliminary research. Moreover, harnessing such learning is essential to ensure that interventions and trial designs are normalised into routine clinical practice [55]. The feasibility and acceptability of a trial designed to measure effectiveness of a VR intervention among women with breast cancer are therefore discussed. In particular, we share learning about recruitment and outcome variables for future effectiveness VR trials and discuss the type of VR intervention required to make a difference among cancer survivors.

### Recruitment

This study shows a range of factors influencing the number of women with breast cancer recruited to the VR trial and number of participants included in final analysis. First, relaxing eligibility criteria may increase the number of women recruited to future trials. Due to the pragmatic nature of this trial and use of an intervention that involved referral to an existing case management VR service, certain eligibility criteria were not under the research team’s direct control. For example, conditions of VR service funding meant that only employees from companies with fewer than 250 employees could be referred until these external restrictions were relaxed. Moreover, challenges associated with integrating recruitment processes that were broadly consistent across three hospital sites and not overly complicated or burdensome dictated the criteria around surgery as first treatment. Finding ways to overcome external factors, such as renegotiating terms of service funding, may increase participation in future RCTs of the effectiveness of existing case management VR services. Second, future multi-centre trials should consider variation in recruitment procedures to accommodate different clinical routines as long as these different procedures do not introduce selection bias. In our study the recruitment process varied marginally in each of the three research sites, accounting for local differences in clinical routines. Comparison of participant characteristics across the three sites did not suggest selection bias. Third, researchers should acknowledge that clinical priorities are likely to supersede those of research, which will influence recruitment rates. In our study clinicians did not approach all eligible patients about the study (31.9% of patients who were eligible were not approached about the study by a clinician) due to competing clinical demands. Any future study involving clinicians (who understandably prioritise clinical demands above those of research) in the recruitment process should therefore factor clinical priorities when calculating recruitment rates. Alternative methods of recruitment that do not involve clinicians in the process should also be considered. Fourth, reasons for non-participation in RCTs of VR services should be examined to increase recruitment to future RCTs. In this study, ethical considerations restricted the ability to ask individuals who chose not to consent to the RCT to identify the reasons for non-participation. A survey of a representative sample of breast cancer patients prior to a future RCT may provide additional evidence to inform effective recruitment strategies. Fifth, the study suggests that age (participants had to be aged <65 years) is a useful inclusion criterion for VR studies because it is a quick method of identifying patients most likely to be eligible, i.e., in paid employment and thus likely to benefit from VR. At the time of the study the average age at which people left the labour market in the UK rose from 63.8 years to 64.6 years for men and from 61.2 years to 62.3 years for women between 2004 and 2010 [57]. Finally, future studies should factor the number of participants eligible for entry into the final analysis when calculating recruitment targets. The study shows that 21.7% of patients were not included in the final analysis for reasons such as failing to complete questionnaires in a timely way or missing items on returned questionnaires.

Thus, taking into account the proportion of patients meeting eligibility criteria, clinical priorities, patient willingness to consent (only a fifth of eligible patients who were given information about the study consented) and the number of participants entered into final analysis, we conservatively estimate that out of every 1,000 patients with breast cancer approximately 1% are likely to be entered into analysis of a VR trial.

### Outcome variables

The study raises several issues about selection of outcome variables for future VR trials.

Our study found that participants had an average of 87.8 days (mean) off work within the first 6 months following diagnosis, which is almost double that found in a North American study, which reported that women with breast cancer had an average of 44.5 days within the first 6 months [19]. This difference in findings may reflect different policies, procedures and economic factors in different countries, which are key variables that impact the transition back to the workplace [58], as well as cultural differences. Our study shows that participants in the intervention group had an average 55.4 days (mean) off work (i.e., closer to duration of work absence reported in the North American study) compared to 108.5 days for participants in the control group. However, this difference in outcome was not statistically significant, which is why we are recommending larger, more definitive effectiveness VR trials that include duration of absence from work as a key outcome variable. Self-reported sick leave was considered appropriate by participants and is a robust method [48,49], which is why we are suggesting that completion of a sick leave questionnaire every 4 weeks is a useful approach.

In relation to other employment-related outcomes, no participants retired, left paid employment or changed jobs during the 12 months post-diagnosis and all participants reported the same job role at 12 months as they had before the cancer diagnosis. A UK study of cancer survivors [59] and studies of breast cancer survivors [14-16] with much larger sample sizes suggest that approximately 20% of cancer survivors will experience one or more of these types of changes in their work pattern. Changes in hours worked were, however, observed in intervention and control group participants but no discernable differences in patterns of change in hours worked between the two groups were observed. A UK study [59] found that 83.2% of cancer survivors taking less than 6 months sick leave were working the same hours as before the cancer diagnosis whereas only 57.1% of those who took 18 months or more sick leave were working the same hours. Thus, hours worked should be considered as an outcome variable in any future effectiveness VR trials, particularly those with a longer term follow-up (i.e., beyond 12 months post-diagnosis).

Participants in the intervention group reported higher overall quality of life at 6-month follow-up (FACT-B: 109.0, SD = 17.9) than those in the control group (98.9, SD = 21.4) but the difference was not statistically significant. Quality of life improved at 12 months for both groups; in the intervention group it increased by nearly 5 points (FACT-B: 113.7, SD 18.5) and by 8 points for women in the control group (FACT-B 107:1, SD 19.8). An observational study of 2,013 women with breast cancer in North America using the same instrument (FACT-B) reported a slightly higher quality of life score (FACT-B: 113.7, SD 18.8) compared to women in our intervention group at 6 months [5]. However, quality of life of women in North America [5] at 6 months and of women in our intervention group at 12 months was identical (FACT-B: 113.7). Given only slight variation in quality of life scores between women in our intervention and control groups (although a note of caution is required because our study was under-powered) and identical quality of life scores to those reported in an observational study, we question whether quality of life, or the specific measure of QoL used, is a sufficiently sensitive outcome for interventions trials. The criterion of sensitivity to change is an important consideration when deciding outcomes for future VR intervention trials [60]. Further, our study shows that some participants found it difficult completing a quality of life questionnaire because their quality of life varied daily (within the first 12 months following diagnosis) whereas the questionnaire requested them to report their quality of life over the previous 7 days.

Cancer-related fatigue has been described as ‘the commonest and most debilitating symptom in patients with cancer’ [61]. A systematic review of 14 studies concluded that there is good evidence that fatigue occurs up to 5 years after completion of adjuvant therapy for breast cancer [62]. Fatigue has been found to predict return to work in cancer survivors [63] and is an important factor related to work productivity in breast cancer survivors even at 3 years post treatment [29]. Nevertheless, our study found only negligible differences in self-reported levels of fatigue (FACIT-Fatigue) between the intervention group and control groups at both 6- and 12-month follow-up that were not statistically significant.

Based on these issues about outcomes highlighted by our study, we propose further research and discussion between key stakeholders (including patients) to determine which outcomes to measure in effectiveness VR intervention trials. Broadly, outcomes must measure important phenomena, be scientifically sound, provide useable information and be feasible to collect [64]. A review of 45 articles on cancer survivors and work, for instance, proposed a work and cancer model where a range of symptoms, functions, and health and well-being categories are associated with work outcomes (e.g., return to work, work ability, work performance and sustainability) [58].

### Intervention

This study evaluated a VR case management service for people requiring work-related support due to ill health. There is no single definition of case management [65]; the Case Management Society of America (CMSA) defines it as ‘a collaborative process of assessment, planning, facilitation, care coordination, evaluation, and advocacy for options and services to meet an individual’s and family’s comprehensive health needs through communication and available resources to promote quality cost-effective outcomes’ [66]. Thus, patients are likely to have a case manager who then refers and directs them to other support services if there is a need.

A comprehensive review of the impact of VR for people with a range of conditions concluded that there is moderate evidence that the use of a case management approach is effective for occupational outcomes [65], although controlled evaluations (a more robust method for measuring effectiveness) of the impact of these interventions were rare and none focused on cancer survivors. A recent controlled before-and-after comparison of a case management intervention for staff in two English hospital trusts found that the proportion of 4-week absences that continued beyond 8 weeks fell from 51.7% to 45.9% in 2 years in the hospital trust receiving the intervention whereas there was an increase from 51.2% to 56.1% in the hospital trust not receiving the intervention – a difference in change of 10.7% (95% CI, 1.5-20.0%) [67]. Thus, based on this evidence there was reason to believe that a case management approach might be appropriate for cancer survivors. This RCT found that women with breast cancer who were referred to a VR service using a case management approach reported 53.1 fewer days of sick leave over the first 6 months post-surgery than those in the control group, although the difference was not statistically significant.

Several reasons may explain why we did not find statistically significant differences between the intervention and control group. First, the study was underpowered; an RCT with a larger sample is therefore required to produce more definitive findings about effectiveness of case management approaches. Second, interviews with BCNs indicate that patients showing signs of anxiety and distress were excluded from the trial. Mental health difficulties have been associated with employment-related outcomes; an observational study of 477 patients with cancer found that 19% were occupationally stressed, which increased their risk of early retirement (odds ratio 5.44) [68]. A relatively healthy participant group may partially explain why only two patients (28.6%) were referred to other services for support. Thus, patients with the greatest level of need may have been excluded from the study, which meant that most participants did not require intensive case management. A third and related factor is that VR staff did not perceive the work as case management because the intervention was considered light touch. Most participants received support over the telephone about work-related issues. This type of support, however, is similar to that provided by other interventions with a work-related component; a review of 19 interventions, 3 of which focussed primarily on improving work outcomes, found that the most frequently reported work-directed component consisted of encouragement, education or advice about work or work-related subjects (68%) [36]. In contrast, a recent before-and-after evaluation of a VR intervention for patients with brain tumours reported that patients received 11 hourly sessions over 5 months that included a broad array of assessment and supportive interventions tailored to individuals’ needs and found a positive impact [69]. Finally, the VR service was not specifically designed to support people with cancer and staff requested further formal training to support cancer survivors. Frequently identified problems following cancer that affect work are fatigue, tiredness and physical limitations [2,7,70], and greater understanding of cancer patients issues, needs and concerns may improve the effectiveness of VR interventions.

## Conclusions

Paid employment is important for a range of reasons including financial security and mental well-being. VR to assist breast cancer patients in the return to work process is therefore an important component of cancer survivorship plans. While there are likely to be health policy and clinical contextual factors influencing study design, conducting a pragmatic trial of effectiveness of a VR intervention among cancer survivors is both feasible and acceptable but more research about the exact components of a VR intervention and choice of outcomes to measure effectiveness is required.

## Competing interests

The authors declare that they have no competing interests.

## Authors’ contributions

GH drafted the protocol, secured funding and ethics approval, conducted data interpretation, and drafted and revised the manuscript. NMG drafted the protocol, secured funding, managed data collection, conducted data analysis and interpretation, and drafted and revised the manuscript. DA provided statistical advice for analysis and review of the manuscript. JMME drafted the protocol, secured funding, and drafted and revised the manuscript. RGK managed recruitment and data collection, conducted data analysis and interpretation, and drafted and revised the manuscript. All authors read and approved the final manuscript.
